# Tracing terahertz plasmon polaritons with a tunable-by-design dispersion in topological insulator metaelements

**DOI:** 10.1038/s41377-025-01884-0

**Published:** 2025-08-26

**Authors:** Leonardo Viti, Chiara Schiattarella, Lucia Sichert, Zhengtianye Wang, Stephanie Law, Oleg Mitrofanov, Miriam S. Vitiello

**Affiliations:** 1 NEST, CNR-Istituto Nanoscienze and Scuola Normale Superiore, Piazza San Silvestro 12, 56127 Pisa, Italy; 2 University of Regensburg, 93040 Regensburg, Germany; 3 Department of Materials Science and Engineering, University of Delaware, Newark, DE 19716 USA; 4 Materials Science and Engineering, Pennsylvania State University, University Park, PA 16802 USA; 5 University College London, Electronic and Electrical Engineering, London, WC1E 7JE UK

**Keywords:** Metamaterials, Microscopy

## Abstract

Collective oscillations of massless charge carriers in two-dimensional materials—Dirac plasmon polaritons (DPPs)—are of paramount importance for engineering nanophotonic devices with tunable optical response. However, tailoring the optical properties of DPPs in a nanomaterial is a very challenging task, particularly at terahertz (THz) frequencies, where the DPP momentum is more than one order of magnitude larger than that of the free-space photons, and DDP attenuation is high. Here, we conceive and demonstrate a strategy to tune the DPP dispersion in topological insulator metamaterials. We engineer laterally coupled linear metaelements, fabricated from epitaxial Bi_2_Se_3,_ with selected coupling distances with the purpose to tune their wavevector, by geometry. We launch and directly map the propagation of DPPs confined within coupled meta-atoms via phase-sensitive scattering-type scanning near-field nanoscopy. We demonstrate that the DPP wavelength can be tuned by varying the metaelements coupling distance, resulting in up to a 20% increase of the polariton wavevector Re(k_p_) in dimers and triplets with a 1 μm spacing, with reduced losses and a >50% increase of the polariton attenuation length.

## Introduction

Surface plasmon polaritons (SPPs)^1^—hybrid light–matter modes involving collective oscillations of mobile charges that propagate along material’s surface^2^—can enable extreme light localization, hence opening interesting prospects in optics, microscopy, sensing, and Raman spectroscopy^3^. They can also modify photon emission rates and be exploited for the development of nanoscale lasers^4^, thanks to the combination of mode confinement to deeply sub-wavelength length scales (down to <1/1000 the free-space wavelength) and enhanced electric field strengths. SPPs have recently been exploited in sub-wavelength plasmonic nanostructures and resonators capable of manipulating light at the nanoscale^5^, an approach particularly effective at far-infrared wavelengths (>60 μm), where plasmonic nanostructures can help circumvent severe limitations imposed by diffraction, enabling sub-diffraction-limit light–matter interaction and enhanced nonlinear phenomena^6^.

Far-infrared sub-wavelength metallic resonators have been implemented as individual elements and as designer metasurfaces for frequency filtering^7^, wavefront shaping^8^, beam steering^9^, polarization rotation^10,11^ and ultra-strong light–matter coupling^12^, albeit without being tuneable. In contrast, scalable two-dimensional (2D) materials^13^, which support deeply sub-wavelength surface plasmon-polariton quasiparticles^2,14^, offer electrical and optical tunability^15^.

Mapping far-infrared SPPs or Dirac plasmon polaritons (DPPs) in 2D materials has become a prominent research area in recent years^16–25^. At THz frequencies, the polariton dynamics is strongly influenced by the properties and density of charge carriers due to intraband absorption (free-carrier response). Furthermore, Dirac materials, such as graphene, Weyl semimetals, and topological insulators (TIs), exhibit low electronic specific heat because of the reduced density of states near the Dirac point^26^. As a result, THz excitation can lead to huge thermal gradients (up to ~1000 K in graphene^27^), leading to non-equilibrium distribution of particles within the band structure with a significant change in the optical conductivity. The latter, in turn, results in an intensity-dependent optical response, i.e., strong optical non-linearity^27,28^.

Topological insulators, such as Bi_2_Se_3_, support THz DPPs at the surface^18,20,24,29–31^ with high momentum, which can be modified via plasmon coupling to phonons and massive charge carriers in the bulk and at the surface^32^. In principle, TIs allow tailoring DPP dispersion properties through proper resonator designs and/or lateral coupling between a set of neighboring TI resonators. Recently, a powerful methodology for investigating DPPs in mechanically cleaved^17^ and large-area Bi_2_Se_3_ samples grown by molecular beam epitaxy (MBE)^18,23^ has been developed using scattering-type scanning near-field optical microscopy (s-SNOM)^33^. More recently, it was applied to detect and characterize hybrid polaritons in Bi_2_Se_3_ antennas, which proved to be effective in confining the propagation of DPPs to a single dimension, thereby enhancing the visibility of DPPs despite the strong intrinsic attenuation^24^.

Here, by using THz-s-SNOM with laser feedback interferometry^34,35^ in THz quantum cascade lasers (QCLs), we reveal a strong effect of inter-resonator coupling in Bi_2_Se_3_ doublet or triplet antennas, and show that the DPP dispersion can be modified by controlling the lateral gap between lithographically defined antennas. We determine dispersion properties of coupled DPPs propagating in the antennas at THz frequencies and show that the inter-resonator coupling leads to an increase of the DPP momentum by over 20%, resulting in values more than one order of magnitude higher than the free-space photon momentum. While the real part of the momentum increases, the loss remains similar to the case of uncoupled resonators, suggesting that the inter-resonator coupling strategy can mitigate the effects of strong DPP intrinsic losses observed in 2D materials^36^. By carefully choosing coupling distances and resonator design, one could enable quantum devices exploiting the properties of DPPs. This result has significant implications for the development of integrated plasmonic devices that rely on sub-wavelength metaelements for manipulating THz radiation. Furthermore, it holds promise for applications in quantum information^37^, spintronics^38^, THz photonics^39^, and laser physics^40^.

## Results and discussion

**Fig. 1:**
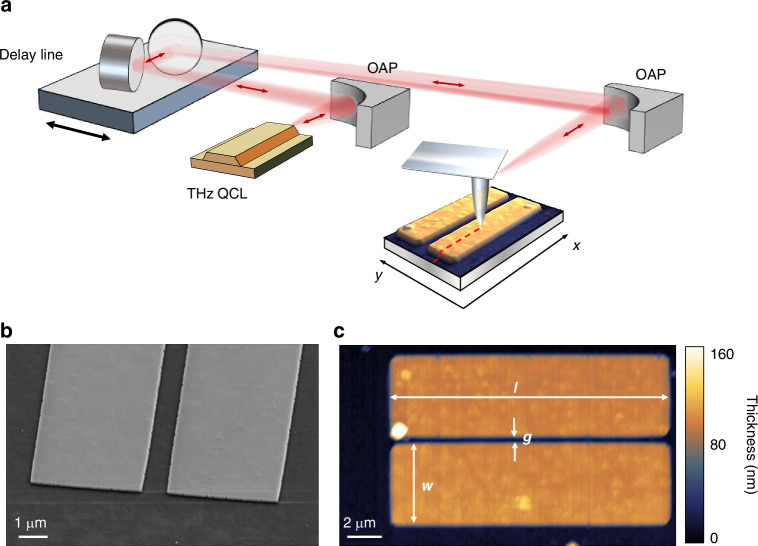
Experimental setup. **a** Schematic of the THz s-SNOM setup in detector-less configuration. The output THz radiation from the QCL is collimated by an off-axis parabolic (OAP) mirror. The optical path length is set by a delay line. The AFM tip tapping frequency is *Ω*~53 kHz. SM signal is measured with a lock-in amplifier as a modulation of the voltage across the QCL terminals. A full description of the setup is given in the Methods section. **b** 30° tilted scanning electron microscope image of one of the fabricated Bi_2_Se_3_ resonators on a sapphire (Al_2_O_3_) substrate. **c** AFM image of a 16 × 4 μm^2^ dimer with 0.5 μm gap. Italic letters *l*, *w*, and *g* indicate the length, width and gap of the coupled resonators, respectively. A full description of the fabrication procedure is given in the Supplementary Information

The THz s-SNOM apparatus, adopted in the present work, operates in a phase-sensitive detector-less configuration through self-mixing (SM) interferometry, with a set of three THz QCLs^34,35^. This method has proven instrumental for launching and capturing the propagation of THz plasmon polaritons in samples with reduced dimensionality, via the real-space mapping of surface waves with wavelengths smaller than *λ*_0_/100^21,41^, where *λ*_0_ is the wavelength of the free-space propagating wave. The laser beam is focused on an atomic force microscope (AFM) tip, which acts as a local plasmon excitation source when positioned above the sample surface. Figure 1a shows a schematic of the experimental setup (see Supplementary Information for further details). The illuminated tip induces a strongly confined polarization in the surface, and thus it can couple the incident beam to a surface-guided polariton with a wavevector much higher than that of the free-space propagating wave. In our Bi_2_Se_3_ resonators, the polariton travels along the antenna, reflects off the antenna ends and returns towards the AFM tip, which then scatters it back to the QCL. The scattered wave couples into the laser cavity and mixes with the field inside the cavity, resulting in a change of the QCL terminal voltage. For a tip tapping near the sample surface (~200 nm distance) with a frequency *Ω*, the terminal voltage therefore displays oscillation at the fundamental frequency and its higher harmonics (n*Ω*), enabling the detection of extremely weak scattered waves, without the need for a THz detector.

This modulation of the QCL voltage, i.e., the near-field SM signal, carries information about the amplitude and phase of the launched DPPs, detectable even at the 5th harmonic frequency^35^. The phase is extracted readily by varying the optical path length *L* between the QCL and the AFM tip, by means of an optical delay line, and recoding an interferometric pattern. The adopted SM detection scheme is inherently fast due to the picosecond lifetime of carriers in QCLs^42^. Therefore, by varying *L* with a constant velocity during the acquisition of a two-dimensional (*x*,*y*) s-SNOM image, it is possible to reconstruct an optical hologram *H*_n_(*x*,*y*) = *A*_n_(*x*,*y*) × exp(*iP*_n_(*x*,*y*)), enabling phase (*ϕ*_n_) and amplitude (*s*_n_) 2D near-field imaging at the *n*th order harmonic $\widetilde{\sigma }$_n_(*x*,*y*) = *s*_n_(*x*,*y*) × exp(*iϕ*_n_(*x*,*y*)) by means of post-processing^43^. Here, we focus on phase detection for accurate measurement of the dispersion of plasmon-polaritons with extremely short propagation lengths^24^. We concentrate our analysis on the third demodulation order, which is chosen as a good compromise between signal intensity and far-field background suppression^34,35^.

The adopted QCL-based holographic approach^43^ enables quantitative retrieval of the plasmon phase, eliminating the need for external photodetectors and interferometric homodyne^44^, heterodyne^45^ or pseudo-heterodyne^46,47^ schemes.

The dispersion of DPPs is influenced by the mobility and the density of electrons in the topological surface states in Bi_2_Se_3_^18,32^. In the THz spectral region, it is also affected by two infrared active phonon modes at 1.92 THz and 4.05 THz^32^, as well as by free massive carriers in the surface and bulk states^18,23^. THz DPPs in Bi_2_Se_3_ have been thoroughly characterized using near-field real-space mapping of plasmons on MBE-grown large-area layers^18,23^ as well as lithographically defined antenna resonators^24^. We selected three single-mode QCLs emitting in the spectral range near the two phonon resonances: at 3.0 THz, 3.4 THz, and 4.3 THz. First, we characterized and verified the dispersion of surface plasmons in a single antenna^18,23,24^, and then we compared the *k*-vector in a doublet and a triplet antenna with the reference single-antenna case.

**Fig. 2:**
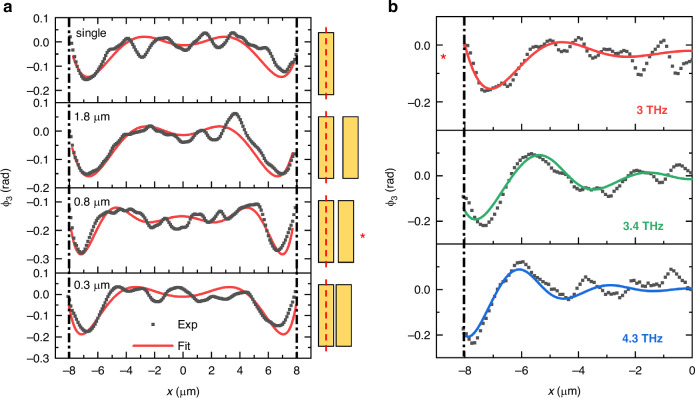
Phase profiles for different configurations and excitation frequencies. **a** s-SNOM SM phase profiles and fitted curves at 3.0 THz for a doublet-antenna resonator with a decreasing gap compared to the single-antenna case (from top to bottom: single, *g* = 1.8, 0.8, and 0.3 μm spacing). **b** Dispersion behavior of the 0.8 μm-spacing doublet-coupled antennas probed at different frequencies: 3.0 THz (the red asterisk marks the correspondence between the two panels), 3.4 THz and 4.3 THz

Our rectangular antennas were designed to be approximately twice as long as the expected DPPs wavelength at 3 THz. For this antenna length, tip-launched DPPs produce at least two full oscillations of SM phase signal *ϕ*_*n*_ along the antenna^24^, sufficient for determining the *k*-vector. We fabricated antennas that are 16 μm long (*l*) and 4 μm wide (*w*). These were etched from an 80 nm thick continuous film of MBE-grown Bi_2_Se_3_ on sapphire (details about MBE growth are provided in the Supplementary Information). Scanning electron microscope (SEM) images and AFM topography of the fabricated coupled resonators are shown in Fig. 1b, c (details about the fabrication procedure are provided in the Supplementary Information). The rectangular antenna geometry, with one dimension much shorter than the other, allows us to confine the polariton propagation to a single dimension, avoiding the geometrical decay^48^ caused by the radial divergence of tip-launched waves. The top panel in Fig. 2a shows the profile of *ϕ*_3_ along a single antenna, where the phase clearly displays an oscillatory behavior starting at the edges and decaying toward the antenna center. The spatial frequency of these oscillations and their decay allowed us to determine real and imaginary parts of the *k*-vector of the tip-launched plasmon by fitting the model of plasmon propagation along the antenna to the experimental data (see Supplementary Information). The results for the single antenna are consistent with previously reported values^24^. By comparing the profile shown in Fig. 2a for a Bi_2_Se_3_ resonator with that obtained from a gold resonator of identical shape (see Supporting Information), we can unveil the presence of structured noise in our experiments. To study how the SPP spatial frequency changes when another antenna of the same geometry is placed near and parallel to the first antenna, we varied the gap (*g*) between antennas in two geometries—in the doublet and triplet antennas. We found that the size of the gap significantly affects the observed phase profile.

Figure 2a shows the results obtained for three different values of doublet gap (*g*), 1.8, 0.8, and 0.3 μm, indicating varying optical coupling. For the 0.3 μm gap in the doublet geometry, the spatial frequency decreased slightly compared to the single isolated antenna case; the spatial frequency remained similar to that retrieved in the single antenna for the 1.8 μm gap. Conversely, for the 0.8 μm gap, the spatial frequency visibly increased. These changes in *ϕ*_3_ profiles along the antenna axis indicate that the plasmon dynamics in the doublet and triplet antenna resonators are strongly influenced by the lateral near-field coupling.

We also considered the case of 0.8 μm gap and used different QCLs to excite plasmons at higher frequencies. Figure 2b shows the *ϕ*_3_ profiles acquired along the doublet resonator for excitation frequencies of 3.0, 3.4, and 4.3 THz. In all cases, the plasmon-polariton *k*-vector (*k*_p_) increased in the doublet compared to the single-antenna case. Indeed, the corresponding wavelength is reduced in the doublets, as evidenced by the comparison between the red fitting curves in Fig. 2a.

**Fig. 3:**
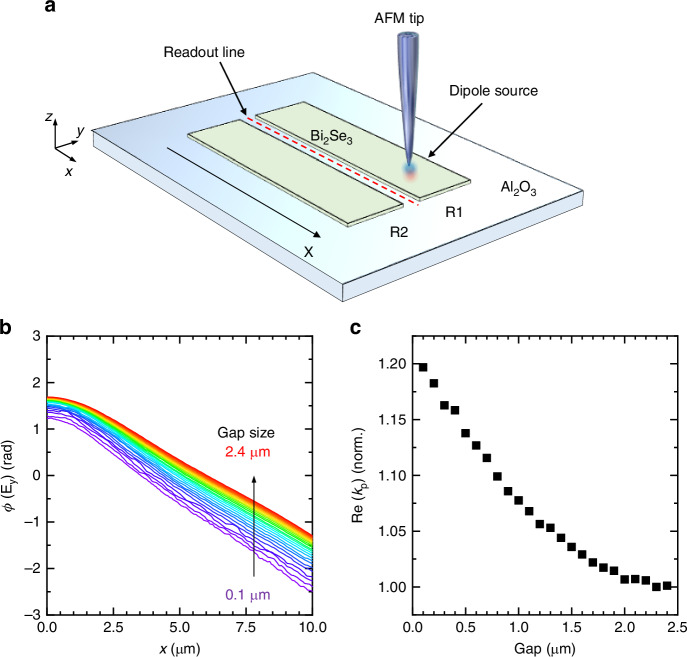
Electromagnetic simulations of plasmon-polaritons launched by the s-SNOM tip in a doublet-antenna resonator. **a** Schematics of the model: a 3.0 THz point dipole source, representing the s-SNOM tip, excites the system above the antenna labeled as R1 (input resonator). The point dipole is positioned above the Bi_2_Se_3_ surface at *z* = 800 nm. The propagating SPP is monitored by a probing line located in the gap between the two resonators at *z* = 0 nm (air-sapphire interface). Resonators are simulated as infinitely thin conductive layers above a 40 μm thick sapphire substrate. **b** Phase of the transverse electric field (*E*_y_) between the two resonators plotted as a function of distance, for gap size *g* ranging from 100 nm to 2.4 μm. **c** Normalized plasmon-polariton momentum (real part) estimated from the slope of the phase profiles in (**b**). A momentum increase of up to 20% is expected when *g* = 100 nm, with respect to the standalone resonator

To gain an understanding of the antenna gap effect, we conducted electromagnetic simulations of plasmon coupling between two long adjacent antennas in the doublet resonator. Electromagnetic simulations were performed using a commercial software based on a finite element method (Comsol Multiphysics^®^^49^, see “Methods” section for further details). In the simulated model (Fig. 3a), one resonator (R1) was excited by a dipole point source, mimicking the excitation produced by the s-SNOM tip. We then observed the propagation of the DPP along the antenna axis. To ensure the DPP propagation over a sufficiently long distance, we modeled the antenna to be 30 μm long, i.e., equal to several plasmon attenuation lengths. By analyzing the in-plane component of the electric field (*E*_y_) in the gap between the antennas, we extracted the plasmon *k*-vector. As the gap (*g*) between the two resonators is decreased from 2.4 μm to 100 nm, we observed that the rate at which the phase of *E*_y_ changes progressively increases (Fig. 3b). This suggests that the *k-*vector increases as the gap becomes smaller. Figure 3c illustrates the relationship between the real part of the wavevector Re(*k*_*p*_) and *g*, showing a progressive decrease in Re(*k*_*p*_) as the resonators are moved further apart.

**Fig. 4:**
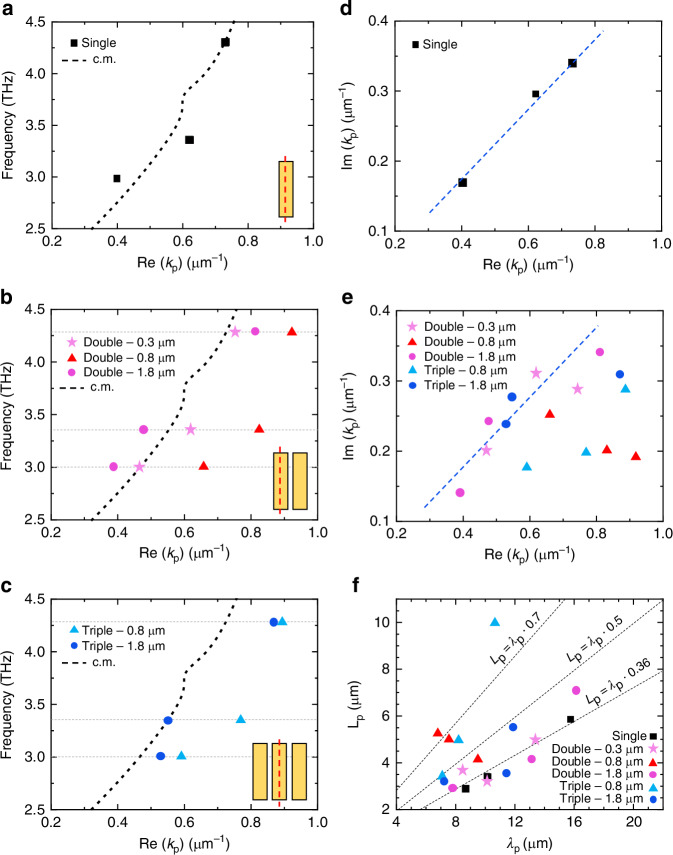
Polariton dispersion for different device configurations. **a** Polariton dispersion obtained by fitting the experimental phase profiles for the single antenna. The black dashed line corresponds to the SPP dispersion predicted by an analytical conductivity model (c.m.), described in the Supplementary Information. **b**, **c** Polariton dispersion for the doublet (**b**) and triplet (**c**) configurations. The black dashed lines correspond to the lines in (**a**), facilitating a direct comparison with the single-antenna case. Horizontal lines indicate the three investigated frequencies: 3.0, 3.4, and 4.3 THz. **d** Imaginary part vs. real part of the polariton wavevector for a standalone resonator. The blue dashed line is a guide to the eye. **e** Imaginary part as a function of the real part of the polariton wavevector in the cases of doublets and triplets. Dashed lines correspond to those in (**d**). **f** Polariton decay length (*L*_p_) as a function of the polariton wavelength (*λ*_p_). The dashed lines represent different proportional relationships between the two quantities. The line *L*_p_ = *λ*_p_ × 0.36 is derived from a fit to the single-resonator measurements

From the analysis of the phase oscillations in Fig. 2 we obtain the complex value of the plasmon-polariton wavevector *k*_p_ = Re(*k*_p_) + *i*Im(*k*_p_), which encodes the polariton propagation characteristics, in particular the polariton wavelength *λ*_p_ = 2*π*/Re(*k*_p_) and its damping length *L*_p_ = 1/Im(*k*_p_). The detailed fitting procedure is described in the Supplementary Information. The results of the analysis are shown in Fig. 4, which displays the extracted dispersion (Fig. 4a–c) and damping (Fig. 4d–f) retrieved for all the measured coupled resonator configurations, at the three frequencies under study: 3.0 , 3.4, and 4.3 THz, indicated as dashed horizontal lines (Fig. 4b, c). Figure 4a displays the measured DPP dispersion for a standalone 16 × 4 μm resonator, demonstrating a wavelength compression ratio (*λ*_0_/*λ*_p_) ~10, consistent with previous reports^18^. The experimental data are compared to the polariton dispersion predicted by an analytical conductivity model^18,21,50^. In this model, the Bi_2_Se_3_ layer is considered as a layer of zero thickness, with a total conductivity (*σ*_t_) given by the sum of bulk (*σ*_bulk_), massive surface carriers (*σ*_2DEG_), and Dirac carriers contributions (*σ*_DC_). The complex polariton wavevector *k*_*p*_ is evaluated as *k*_*p*_ = i*k*_*0*_*ε*_r_ × (*c*/2π*σ*_t_)^18^, where *k*_*0*_ is the free-space wavevector, *ε*_r_ = (*ε*_sub_ + *ε*_sup_)/2 is the average permittivity of the materials surrounding the Bi_2_Se_3_ layer (substrate and superstrate), and *c* is the speed of light. The best agreement between the experimental data points and the analytical model is achieved with the following values of carrier densities: *n*_bulk_ = 1.8 × 10^19 ^cm^−3^, *n*_DC_ = 1.0 × 10^13 ^cm^−2^, and *n*_2DEG_ = 0.4 × 10^13 ^cm^−2^, in good agreement with previous reports on similar structures^24^. Further details on the analytical model and on the evaluation of the three conductivities, *σ*_bulk_, *σ*_2DEG_, and *σ*_DC_, are provided in the Supplementary Information.

We then investigated how the dispersion of DPPs is modified when the Bi_2_Se_3_ antennas are laterally coupled in dimers or triplets. Figures 4b, c display the obtained results, indicating that the DPP’s dispersion is notably different when the resonators are separated by a gap *g* = 0.8 μm. Specifically, the polariton wavevector Re(*k*_p_) is increased by over 20% in dimers and triplets with this gap, compared to the predicted trend (dashed lines in Figs. 4b, c) for the case of standalone resonators. Interestingly, the coupling strength *η*, quantified here as the percentage increase of Re(*k*_p_) relative to the standalone resonator, exhibits a non-monotonic dependence on the gap *g* between the two coupled resonators: *η* has a maximum value for *g* = 0.8 μm. We attribute this observation to the fact that, for *g* = 0.3 μm, the separation between two neighboring antennas is small enough that the SPP is transmitted through the gap with negligible reflection.

The effect of lateral coupling can also be observed in the analysis of the DPP propagation length *L*_p_. Figure 4d presents the Im(*k*_p_) values plotted against Re(*k*_p_) for all QCL frequencies for the standalone resonator. In this configuration, the ratio Re(*k*_p_)/(2*π* × Im(*k*_p_)) = *L*_p_/*λ*_p_, which represents the relative attenuation length of the SPP, remains almost constant regardless of frequency at approximately 0.36.

This value is consistent with that reported in previous studies on MBE-grown Bi_2_Se_3_ thin films^24^ and can be attributed to a smaller wavelength compression ratio *λ*_0_/*λ*_p_ compared to other material systems, such as hBN-encapsulated graphene. Interestingly, the relative attenuation length is significantly improved in efficiently coupled resonators, as shown in Fig. 4e, which plots Im(*k*_p_) as a function of Re(*k*_p_) for various configurations of coupled resonators. In this figure, data points to the right of the diagonal dashed line represent configurations with lower attenuation. This observation can be further clarified by visualizing the same results in the (*L*_p_,*λ*_p_) plane (see Fig. 4f), where we observe that for a gap spacing of *g* = 0.8 μm and frequencies above 3.4 THz, the relative attenuation length *L*_p_/*λ*_p_ is approximately doubled with respect to that of the standalone antenna, indicating that the propagation losses are strongly reduced in laterally coupled systems. This loss reduction can be intuitively understood as a result of decreased lateral confinement of the propagating SPP along the antenna, due to the evanescent coupling with neighboring structures. However, when the confinement is further reduced (*g* = 0.3 μm), the surface mode can spread across the separation gap, resulting in standard geometrical damping and canceling out the benefits of the *quasi*-one-dimensional propagation. For larger gap spacing (*g* = 1.8 μm), the coupling between neighboring resonators is strongly reduced, leading to propagation losses similar to those observed for the standalone antenna.

## Conclusions

In this study, we demonstrate that the wavevector of plasmon polaritons on the surface of Bi_2_Se_3_ resonators can be tuned by selecting the gap between laterally coupled resonators. These have been designed and fabricated from large-area MBE-grown Bi_2_Se_3_ thin films. The propagation of plasmon polaritons on the resonator surface has been characterized using THz detector-less s-SNOM, enabled by laser feedback interferometry in QCLs. Near-field measurements allow us to estimate the complex polariton wavevector at 3.0, 3.4, and 4.3 THz. The investigation of tip-launched SPPs propagating on the TI surface shows that the gap (*g*) between adjacent resonators plays a crucial role in determining the polariton dispersion and dynamics. Importantly, *g* not only affects the wavelength but also influences the damping losses of the SPP modes excited on the TI surface. Our results demonstrate that it is possible to customize the spectral response of Bi_2_Se_3_-based THz resonators by adjusting the gap. This knowledge can be adopted as a design strategy for the implementation of TI-based architectures, taking advantage of various effects such as resonance line narrowing, resonance mode-splitting, and cross-polarization conversion, typically observed in coupled resonator systems. Additionally, we envision that lithographically designed THz metasurfaces using large-area TI materials with specific spectral responses could have a significant impact on far-infrared optoelectronics and nonlinear nanophotonics.

## Materials and methods

### Electromagnetic simulations

Electromagnetic simulations were performed using commercial software (COMSOL Multiphysics^®^), based on a finite element method. The resonators in the simulation geometry are long enough to contain multiple plasmon polaritons wavelengths and to be much longer than the polaritons damping length (30 × 4 μm). They are modeled as infinitely thin conductive surfaces^50^ located on the substrate (*xy*) plane, with sheet conductivity *σ*_2D_ ranging from 10^4^ to 10^5^ S m^−1^. We observed some variation in the results using different sheet conductivity values *σ*_2D_ (data in Fig. 3b are obtained for *σ*_2D_ = 10^5^ S m^−1^). The sapphire substrate was 40 μm thick, with dielectric constant *ɛ*_Al2O3_ = 10.4, with the surface plane defined as *z* = 0 nm. Surface plasmon in the electromagnetic simulations were generated by a point dipole source (frequency 3.0 THz) aligned along the out-of-plane direction located *z* = 800 nm above the resonator surface (input resonator, R1). The propagation of the SPP was analyzed by extracting the transverse component of the electric field *E*_y_ at *z* = 0 nm in *xy*-plane. In the case of Fig. 3, we monitor the distribution of *E*_y_ along the *x*-axis at the center of the gap between the two resonators.

## Supplementary information

Supplementary Information

## Data Availability

The data that support the plots within this paper and other findings of this study are available from the corresponding authors upon reasonable request.

## Code availability

The codes and simulation files that support the plots and data analysis within this paper are available from the corresponding author upon reasonable request.
