# Supplementary material for: Tracing terahertz plasmon polaritons with a tunable-by-design dispersion in topological insulator metaelements
Source: Light Sci Appl. 2025 Aug 26;14:288. doi: 10.1038/s41377-025-01884-0 (PMC12381016; doi:10.1038/s41377-025-01884-0)
Supplement: Supplementary file 1 — Supplementary Information [file 41377_2025_1884_MOESM1_ESM.docx]

**Supporting Information**

**Tracing terahertz plasmon polaritons with a tunable-by-design dispersion in topological insulator metaelements**

**Leonardo Viti,^1*^ Chiara Schiattarella,^1*^ Lucia Sichert,^1,2^ Zhengtianye Wang,^3^ Stephanie Law,^4^
Oleg Mitrofanov,^5^ and Miriam S. Vitiello ^1^**

*^1^NEST, CNR-Istituto Nanoscienze and Scuola Normale Superiore, Piazza San Silvestro 12, 56127 Pisa, Italy*

*^2^University of Regensburg, 93040 Regensburg, Germany*

*^3^Department of Materials Science and Engineering, University of Delaware, Newark, Delaware, 19716 USA*

*^4^Materials Science and Engineering, Pennsylvania State University, University Park, PA 16802 USA*

*^5^University College London, Electronic and Electrical Engineering, London WC1E 7JE, UK*

**Authors contributed equally to this work*

**S1- Near-Field Optical Microscopy**

THz QCLs were mounted in a liquid helium continuous-flow cryostat (Janis tech.) with optical access through a high-density polyEthylene (HDPE) window and kept at a fixed heat-sink temperature of 12 K. Temperature stability of 0.2 K was required for phase stability of the self-mixing signal. A diverging THz beam emitted by the QCL is collimated using a 90° off-axis parabolic mirror (OAP) with a focal length of 50 mm. The collimated THz beam passed through an optical delay-line, equipped with two 45 deg, 2 inches plane mirrors mounted on a linear translation stage (Physik Instrumente, stepper motor stage M403.62S) with a 30 nm precision. The beam was then fed into the entrance optical port of a commercial near-field microscope (NeaSNOM, Neaspec/attocube, Martinsried, Germany). A second OAP with a focal length of 25 mm focused the *p*-polarized THz beam onto the AFM tip of the SNOM (25PtIr300B-H40, Rocky Mountain Nanotechnology, tip radius 40 nm, shank length 80 μm) [1]. THz radiation back-scattered by the tip-sample interaction was collected by the same focusing optics and coupled back into the QCL cavity along the same incident optical path. Background (far-field) noise was suppressed by demodulating the SM signal at higher harmonics of the dithering tip frequency (*Ω*~53 kHz), using a lock-in detection scheme. Here, we chose to analyse the 3^rd^ harmonics (3*Ω*) of the fundamental frequency. The voltage modulation across the QCL terminals produced by the self-mixing effect was pre-amplified by a low-noise voltage pre-amplifier (DL Instruments, mod. 1201, gain 200, bandwidth 400 kHz), before lock-in demodulation.

To reconstruct the holograms, one-dimensional H_3_ profiles were acquired by scanning the AFM tip iteratively over the same path along individual antennas, with a 100 nm sampling step. At the beginning of each scan, the optical path length (*L*) between the QCL and the AFM tip is varied by moving the delay-line of a 5 μm step, which corresponds to Δ*L* = 20 μm. This value is chosen to allow the retrieval of frequencies up to the 3^rd^ harmonics of the fundamental QCL frequency (ω_0_) after the Fourier transform of the hologram (Nyquist-Shannon sampling theorem [2]). The delay line was moved by 256 steps, covering approximately 1 mm distance. The Fourier transform of the interferogram provides the amplitude (s_3_) and phase (ϕ_3_) spectra of the s-SNOM signal (σ_3_) for every point along the scanned antenna axis. By frequency-filtering around ω_0_, we obtain amplitude and phase profiles of the near-field signal at ω_0_.

**S2- Sample growth and fabrication**

**MBE growth of Bi_2_Se_3._** The Bi_2_Se_3_ film was grown using a Veeco GENxplor molecular beam epitaxy system on an as-shipped epi-ready 10 mm × 10 mm × 0.5 mm *c*-plane sapphire substrate. The substrate is first baked in a load lock (pressure less than 10^-7^ Torr) at 200 °C for 12 hours and then transferred to the growth chamber (system base pressure < 10^-9^ Torr). Before growing the samples, the substrate was degassed by heating to 650 °C with a 20 °C/min ramp rate and held at 650 °C for 5 minutes, while monitoring its temperature with a non-contact thermocouple. The substrate is then cooled to 325 °C. The Bi_2_Se_3_ thin film was grown via a two-step method: a 4 nm Bi_2_Se_3_ seed layer was first deposited at 325 °C and then annealed at 425 °C for 20 min; the remaining 76 nm of the film was then deposited at 425 °C using co-deposition with a growth rate of 0.6 nm/min. Selenium was supplied using a cracker source, while bismuth was supplied using a dual-filament source. After the deposition is complete, the film was cooled to 200 °C under a selenium flux and transferred out of growth chamber once the substrate temperature dropped below 200 °C. The film was then vacuum sealed for storage before further processing.

**Fabrication.** Bi_2_Se_3_ resonators were fabricated from the uniform 80 nm – thick film with a *top-down* method, by electron beam lithography (EBL, 20 kV). For this purpose, a two-layer resist composed by a positive poly(methilmetacrilate) (PMMA, 300 nm), capped by a conductive coating (AR-PC 5090), is employed, to avoid charging effects over the insulating sapphire substrate. The conductive coating was removed by dipping the sample in deionized water for 2’ before developing exposed PMMA in a solution of methyl-isobutyl-ketone and isopropanol (1:3, 3’). The patterned resonators were then metallized with a thin (20 nm) aluminium layer, which serves as a hard mask for the subsequent etching step. The unprotected regions of the 80 nm Bi_2_Se_3_ film were etched away by dry reactive-ion-etching (RIE), using a Ar plasma (3’, 50 W, etch rate ~30 nm/min), to expose the sapphire substrate. The Al hard mask was eventually removed by selective wet-etching in a solution of Tetramethylammonium hydroxide and water (<5% v/v, MF^®^319 Microposit^®^, 90ʺ, room temperature).


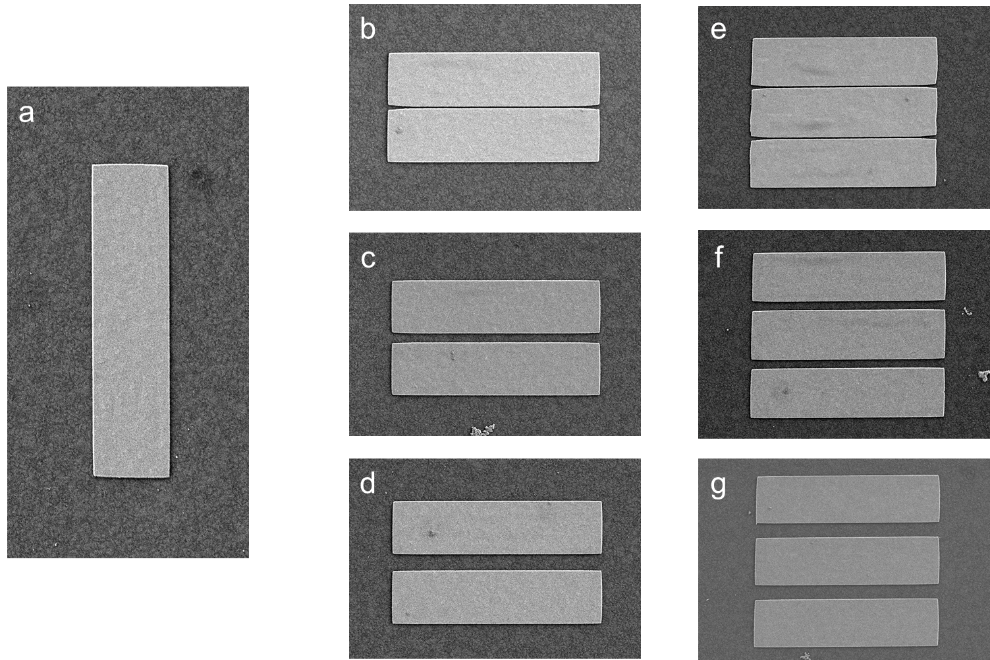


**Figure S1. Scanning Electron Micrographs of resonators. (a)** Single 16×4 μm^2^ resonator. **(b-d)** Doublets with gap distance 0.3 μm, 0.8 μm and 1.8 μm. **(e-g)** Triplets with gap distance 0.3 μm, 0.8 μm and 1.8 μm.

**S3-Near-field phase profiles fitting**

From the analysis of the phase oscillations along the antenna length, we obtained the complex-value of the polariton wavevector *k*_p_ = Re(*k*_p_) + *i*Im(*k*_p_), determining the polariton wavelength 𝜆_p_ = 2𝜋/Re(*k*_p_) and the attenuation length *L*_p_ = 1/Im(*k*_p_) experimentally.

The model used to fit the data assumes that the near-field self-mixing signal (σ_3_) along the antenna (*x*-axis), contains the surface plasmon-polariton contribution due to the tip-launched SPPs, which can be mathematically described by the following expression [3,4]:

$E_{p}\left( x,y \right)=\boldsymbol{A}\left( e^{i2k_{p}\left( x+\frac{l}{2} \right)}+e^{-i2k_{p}\left( x+\frac{l}{2} \right)} \right)+$**A**_+_(*y*)+**A**_−_(*y*) (1)

Equation (1) describes the superposition of four waves that are counter propagating along the *x-*axis (first term) and *y*-axis (second and third terms). Here, *l* is the antenna length, *x* = 0 represents the centre of the antenna, *A* is the complex amplitude of the SPP propagating along the *x-*axis, and *A*_±_ are the complex amplitudes of two SPPs propagating along the *y*-axis. Importantly, equation (1) does not include the typical geometrical decay term, proportional to 1/√2*x* [3], which arises from the energy conservation of a wave launched by and radially diverging from the tip apex in the absence of lateral material boundaries.

Our fitting strategy relies on minimizing the standard deviation SD, which is defined as follows.

$$SD=\sqrt{\frac{\sum_{n=1}^{N} \left( \phi_{3_{n}}^{(exp)}-\phi_{3_{n}}^{(model)} \right)^{2}}{N}}$$

Here, *N* is the number of total data points or phase samples along the antenna length. In order to avoid the involvement of possible artifacts induced by the edges of the sample and the tip lateral face, we shift both the data interval extremes for fitting by three pixels (= 300 nm) inside the antenna. Since the complex amplitude ***A*** of the SPP wave, which depends on the material optical response, is unknown and can vary with the incident frequency, we allow ***A*** to vary when determining the *k*-vector values. To select the best fit, we initially varied the complex amplitude ***A*** (both its modulus and phase) and calculated the standard deviation SD for each set of Re(*k*_p_) and Im(*k*_p_). The initial guess for the fitting curve therefore corresponds to the combination of values that minimized SD. We then fixed the corresponding *optimum* complex amplitude **A*** and re-iterated the SD minimization to determine the optimal Re(*k*_p_) and Im(*k*_p_). The accuracy in determining the optimum *k*_p_ using this approach is limited by the width of the SD surface minimum. **Figure S2** shows the method used to determine the uncertainty over the evaluated Re(*k*_p_) and Im(*k*_p_) from the fitting procedure. The cross-section of the SD surface has a minimum in both the Re(*k*_p_) and Im(*k*_p_) coordinates. By increasing SD of the 5% with respect to the minimum, we obtain a certain *width* of the SD surface, which we considered a good estimation of the error on the fitting parameter. With this method, we obtained a relative uncertainty ΔRe(*k*_p_)/ Re(*k*_p_) < 15% for all the investigated datapoints. We attribute this limitation in accuracy to the strong damping of SPPs in Bi_2_Se_3_, which unavoidably reduces a meaningful *x* interval within the phase spatial profile and thereby increases the uncertainty of *k*_p_.


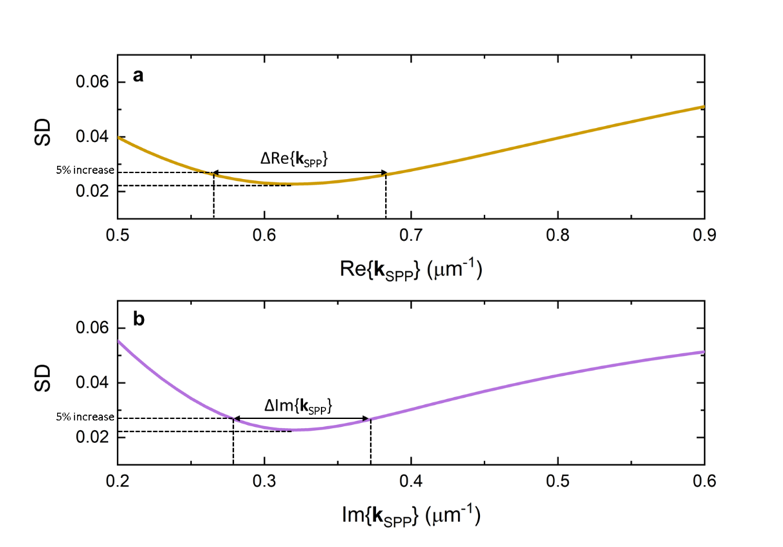


**Figure S2. (a)** Cross-section of the SD surface plotted as a function of Re(k_p_). **(b)** Cross-section of the SD surface plotted as a function of Re(k_p_). Plots are obtained from the fit to the data relative to the single antenna, measured at 3.4 THz.

**S4 - THz s-SNOM holography applied to a gold resonator**

In this section, we report on the measurement of the near-field s-SNOM signal acquired on a gold resonator 16 μm ×4 μm, ~70 nm thick, realized on a silicon substrate by electron beam lithography and metal evaporation. **Figure S3a** shows the scanning electron microscope image of the resonator. **Figure S3b** displays the height (Z) profile of the gold resonator, together with the 3^rd^ harmonic amplitude (s_3_) and phase (ϕ_3_) signals acquired by the THz s-SNOM experiment under illumination with a 3.4 THz frequency. We clearly see no phase or amplitude oscillations along the resonator, testifying the absence of structured noise in our experimental configuration.


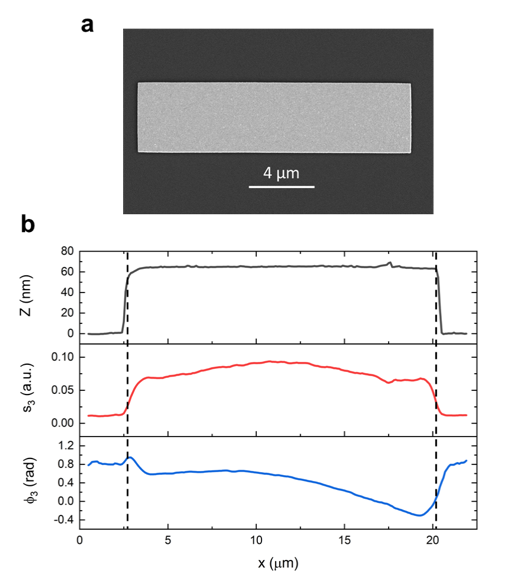


**Figure S3. (a)** Scanning electron microscope (SEM) image of a *~*70 nm thick gold resonator, with dimensions 16 μm × 4 μm. **(b)** Topography (Z), 3rd harmonic near-field amplitude (s_3_) and phase (ϕ_3_) measured with an excitation frequency of 3.4 THz.

**S5 - Amplitude distribution of coupled Bi_2_Se_3_ antennas extracted via THz s-SNOM holography**

***
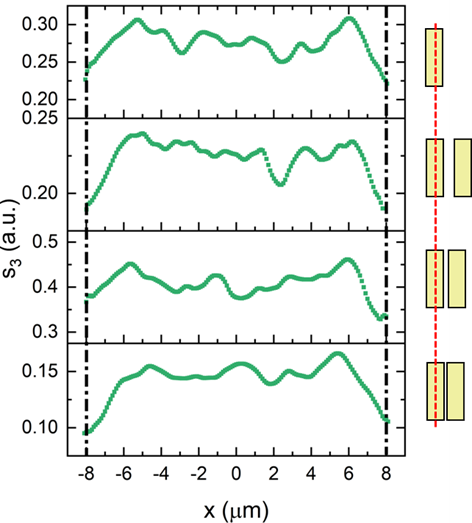
***

***Figure S4.*** *Third-order demodulated near-field amplitude spatial profiles s_3_(x) of the 16x4 µm^2^ antenna doublet set at different spacings, corresponding to the phases φ_3_(x) reported in Fig. 2a.*

**S6 – Analytical model for polariton dispersion on topological insulators**

The conductivity model (c.m.) has been successfully employed in various s-SNOM studies to describe the expected propagation of surface polariton modes on thin slabs of conductive materials [3-5]. In particular, it combines the influence of different types of carriers (e.g., surface Dirac carriers, bulk carriers, etc.), taking advantage of the superposition of the individual conductivities. Moreover, it conveniently describes both in-plane isotropic [3,4] and anisotropic [5] materials.

In this study, we use the conductivity model to evaluate the SPP dispersion in the frequency range between 2.5 THz and 4.5 THz for a Bi_2_Se_3_ thin film (thickness: 80 nm). This model treats Bi_2_Se_3_ as an infinitesimally thin conducting material, an assumption that avoids the calculation of fields inside the material, and that is justified when the material thickness is much smaller than the polaritons wavelength. The total sheet conductivity σ_TI_, is determined by the interplay of contributions from the bulk (σ_bulk_), the topological (Dirac) surface states (σ_DC_), and the massive two-dimensional electron gas (σ_2DEG_) that arises from the downward bending of the conduction band at the surface [3].

The bulk conductivity is calculated using the expression [6] σ_bulk_ = ε_bulk_·(c·d/2iλ_0_), where λ_0_ = 2π/k_0_ is the free-space wavelength and d is the thickness of the Bi_2_Se_3_ film. The frequency-dependent complex permittivity ε_bulk_ is evaluated by considering only the in-plane term, ε^⊥^_bulk_, and neglecting the contribution from the out-of-plane part of the permittivity tensor, ε^||^_bulk_, using a standard Drude-Lorentz model [1,3,4].

The surface conductivity of Dirac carriers is approximated by the expression [3]:

$$\sigma_{\mathrm{DC}}=\frac{e^{2}k_{B}T\cdot ln\left[ 2cosh\left( \frac{E_{F}}{2k_{B}T} \right) \right]}{2\hbar^{2}\pi}\cdot\frac{i}{\omega}$$

where k_B_ is the Boltzmann constant, T is the temperature, and E_F_ is the Fermi energy given by E_F_^2^ = (ℏv_F_)^2^·4πn_DC_. In this expression, ℏ is the reduced Planck’s constant, v_F_ = 5×10^8^ cm/s and n_DC_ is the density of topologically protected (Dirac) surface states.

The conductivity of massive surface carriers is approximated by the expression [3]:

$$\sigma_{2DEG}= \frac{e^{2}n_{2DEG}}{m^{*}}\cdot\frac{i}{\omega}$$

where n_2DEG_ is the massive carrier concentration at the surface.

The total conductivity, σ_t_, is eventually calculated as σ_t_ = σ_bulk_ + 2σ_DC_ + 2σ_2DEG_, and the dispersion relation Re(k_p_(ω)) can then be evaluated using the carrier densities 𝑛_bulk_, n_DC_ and n_2DEG_ as fitting parameters.

**References**

[1] V. Pistore, C. Schiattarella, L. Viti, T. Siday, M. B. Johnston, O. Mitrofanov, M. S. Vitiello. Near-field probes for sensitive detectorless near-field nanoscopy in the 2.0–4.6 THz range. Appl. Phys. Lett. 27 May 2024; 124 (22): 221105. <https://doi.org/10.1063/5.0179714>

[2] A. J. Jerri. The Shannon sampling theorem—Its various extensions and applications: A tutorial review. Proceedings of the IEEE, 65, 1565-1596, (1977). doi: 10.1109/PROC.1977.10771

[3] S. Chen, A. Bylinkin, Z. Wang, et al. Real-space nanoimaging of THz polaritons in the topological insulator Bi2Se3. Nat Commun 13, 1374 (2022). <https://doi.org/10.1038/s41467-022-28791-x>

[4] V. Pistore, L. Viti, C. Schiattarella, Z. Wang, S. Law, O. Mitrofanov, M. S. Vitiello. Holographic Nano-Imaging of Terahertz Dirac Plasmon Polaritons in Topological Insulator Antenna Resonators. Small 20, 2308116 (2024). <https://doi.org/10.1002/smll.202308116>.

[5] S. Chen, P. L. Leng, A. Konečná, et al. Real-space observation of ultraconfined in-plane anisotropic acoustic terahertz plasmon polaritons. Nat. Mater. 22, 860-866 (2023).

[6] A. Y. Nikitin, in *World Scientific Handbook of Metamaterials and Plasmonics. Recent Progress in the Field of Nanoplasmonics* Vol. 4 (ed Aizpurua, J.) (World Scientific, 2017).
